# Multi-Phasic CECT Peritumoral Radiomics Predict Treatment Response to Bevacizumab-Based Chemotherapy in RAS-Mutated Colorectal Liver Metastases

**DOI:** 10.3390/bioengineering13020137

**Published:** 2026-01-24

**Authors:** Feiyan Jiao, Yiming Liu, Zhongshun Tang, Shuai Han, Tian Li, Yuanpeng Zhang, Peihua Liu, Guodong Huang, Hao Li, Yongping Zheng, Zhou Li, Sai-Kit Lam

**Affiliations:** 1 Department of Biomedical Engineering, The Hong Kong Polytechnic University, Hong Kong SAR, China; feiyan.jiao@connect.polyu.hk (F.J.); yongping.zheng@polyu.edu.hk (Y.Z.); 2 Department of Gastrointestinal Surgery, General Surgery Center, Zhujiang Hospital, Southern Medical University, Guangzhou 510282, China; lyiming2001@163.com (Y.L.); tangzhongshun2000@163.com (Z.T.); gzhanbo0624@smu.edu.cn (S.H.); 13711486773@163.com (P.L.); hguodong0332@163.com (G.H.); lihaosmuzhujiang@163.com (H.L.); 3 Department of Health Technology and Informatics, The Hong Kong Polytechnic University, Hong Kong SAR, China; litian.li@polyu.edu.hk; 4 Department of Medical Informatics, Nantong University, Nantong 226001, China; y.p.zhang@ieee.org; 5 Research Institute for Smart Ageing, The Hong Kong Polytechnic University, Hong Kong SAR, China

**Keywords:** computed tomography, radiomics, colorectal cancer, metastasis, bevacizumab, treatment response prediction, machine-learning

## Abstract

This study aims to investigate the predictive value of pre-treatment multi-phasic contrast-enhanced computed tomography (CECT) radiomic features for treatment resistance in patients with rat sarcoma virus (RAS)-mutated colorectal liver metastases (CRLMs) receiving bevacizumab-based chemotherapy. Seventy-three samples with RAS-mutated CRLMs receiving bevacizumab-combined chemotherapy regimens were evaluated. Radiomic features were extracted from arterial phase (AP), portal venous phase (PVP), AP-PVP subtraction image, and Delta phase (DeltaP, calculated as AP-to-PVP ratio) images. Three groups of radiomics features were extracted for each phase, including peritumor, core tumor, and whole-tumor regions. For each of the four phases, a two-sided independent Mann–Whitney U test with the Bonferroni correction and K-means clustering was applied to the remnant features for each phase. Subsequently, the Least Absolute Shrinkage and Selection Operator (LASSO) algorithm was then applied for further feature selection. Six machine learning algorithms were then used for model development and validated on the independent testing cohort. Results showed peritumoral radiomic features and features derived from Laplacian of Gaussian (LoG) filtered images were dominant in all the compared machine learning algorithms; NB models yielded the best-performing prediction (Avg. training AUC: 0.731, Avg. testing AUC: 0.717) when combining all features from different phases of CECT images. This study demonstrates that peritumoral radiomic features and LoG-filtered pre-treatment multi-phasic CECT images were more predictive of treatment response to bevacizumab-based chemotherapy in RAS-mutated CRLMs compared to core tumor features.

## 1. Introduction

Colorectal cancer (CRC) represents a malignancy characterized by substantial heterogeneity, encompassing diverse genetic and molecular subtypes [1]. It persists as a leading contributor to cancer-associated deaths [2]. The liver is the most common site of involvement in metastatic CRC [3,4]. Given this clinical reality, the detection and characterization of hepatic metastases rely principally on cross-sectional imaging modalities, including contrast-enhanced ultrasonography, computed tomography (CT), magnetic resonance imaging (MRI), and fluorodeoxyglucose positron emission tomography (FDG-PET) [5]. Metastases portend a dismal prognosis, with 5-year survival rates approaching zero in diffuse disease [6].

Within the therapeutic landscape of metastatic colorectal cancer (mCRC), bevacizumab—a humanized monoclonal antibody targeting vascular endothelial growth factor-A (VEGFA)—holds established clinical utility. The rationale for its use is based on the understanding that metastatic growth critically depends on VEGFA-driven sprouting angiogenesis for vascularization, and radiological imaging plays a pivotal role in managing these liver metastases, guiding detection, staging, treatment planning, and response evaluation [7]. The established paradigm that metastatic growth critically depends on VEGFA-driven sprouting angiogenesis for vascularization provided the foundational rationale for pharmacologically targeting this pathway. This mechanistic insight was directly exploited through the development of bevacizumab, a humanized monoclonal antibody designed to neutralize circulating VEGFA. By binding and sequestering VEGFA, bevacizumab functionally disrupts its interaction with endothelial cell surface receptors, thereby attenuating pro-angiogenic signaling cascades essential for new vessel sprouting and tumor neovascularization [3,4]. However, the efficacy of anti-angiogenic therapies like bevacizumab is intricately linked to the tumor microenvironment (TME) of CRC, which influences cellular composition and signaling pathways governing tumor response [8]. The integration of bevacizumab with conventional cytotoxic chemotherapy constitutes a standard regimen for mCRC, owing to demonstrable improvements in progression-free and overall survival (OS) outcomes [9,10]. Molecular alterations occurring in Kirsten rat sarcoma virus (KRAS) and neuroblastoma RAS viral oncogene homolog (NRAS) significantly worsen disease prognosis. Specifically, RAS mutations confer more aggressive tumor biology and are associated with shorter OS [11]. In mCRC patients with RAS mutation, a bevacizumab regimen combined with chemotherapy (FOLFOX, FOLFIRI, or CAPEOX) is the standard first-line treatment [12]. Despite its established role, optimal patient selection for bevacizumab remains a significant clinical challenge, particularly in differentiating low tumor burden (LTB) from oligometastatic disease for individualized therapeutic approaches. Critically, the efficacy prediction for bevacizumab in LTB colorectal liver metastases (CRLMs) remains a highly understudied area, representing a key unmet clinical need [13]. Moreover, the survival benefit remains modest, extending life expectancy by only months, and intrinsic resistance mechanisms are incompletely characterized. Emerging evidence implicates vessel co-option as a fundamental resistance pathway in CRLMs. This non-angiogenic process enables tumor cells to circumvent VEGF-dependent angiogenesis by infiltrating hepatic plates and co-opting pre-existing sinusoidal vasculature [14].

Emerging research reveals that differential miRNA expression profiles—including miR-92b-3p, miR-10a-5p, and miR-125a-5p—distinguish bevacizumab responders from non-responders in mCRC [15]. Key methodological challenges in miRNA research encompass the following: I. Biological matrix-dependent variability, where miRNA profiles diverge significantly across sample sources (e.g., serum, plasma, whole tissues, cultured cells). Plasma is established as the preferred matrix over serum due to artifactual alterations in miRNA expression induced by coagulation processes during clotting; II. Technical inconsistencies in isolation and analysis, arising from variations in RNA extraction protocols (e.g., commercial kit performance), cDNA synthesis methods, and library construction strategies for high-throughput sequencing, which collectively introduce analytical bias; III. Molecular detection limitations imposed by the short sequence length of mature miRNAs (typically 18–25 nucleotides), complicating the design of specific primers for RT-qPCR assays and increasing risks of cross-hybridization in multiplexed platforms; IV. Functional validation complexities, wherein accurate identification of miRNA-mRNA interactions necessitates multi-level computational prediction followed by extensive experimental verification, which is significantly challenged by the high false-positive rates inherent in computational target predictions [16].

Given the challenges with molecular biomarkers, non-invasive imaging-based approaches offer a promising alternative. Radiomics involves the high-throughput extraction of quantitative features from standard-of-care medical images like contrast-enhanced CT (CECT) and is particularly well-suited for this task. Beyond oncology, radiomics has also demonstrated predictive value in benign hepatic conditions, for instance, in assessing liver parenchyma prior to TIPS creation [17], highlighting the versatility of these computational methods in hepatic imaging. By capturing subtle patterns of tumor heterogeneity, radiomics can non-invasively probe the tumor phenotype. Indeed, machine learning models leveraging CT features have successfully predicted treatment response and aspects of tumor angiogenesis in other hepatic malignancies [18], demonstrating the potential of this methodology.

However, a key limitation of many studies is their focus on the tumor core alone. The efficacy of an anti-angiogenic agent like bevacizumab is critically dependent on the tumor microenvironment, particularly at the tumor-host interface. We hypothesize that the peritumor region, a zone of intense biological activity including neovascularization, holds the most crucial information for predicting response. Therefore, the present study aims to develop and validate a machine learning model based on multi-phasic CECT radiomic features extracted from the peritumor region to identify early resistance to bevacizumab-based chemotherapy in patients with RAS-mutated CRLMs to guide clinical decision-making.

## 2. Materials and Methods

### 2.1. Study Design

This study was to evaluate the predictive value of pretreatment multi-phasic CECT-based radiomic features for bevacizumab-based chemotherapy resistance in RAS-mutated CRLMs. Patients who were diagnosed with histologically confirmed colorectal adenocarcinoma and received chemotherapy with bevacizumab combined with standard regimens, including FOLFOX, FOLFIRI, and CAPEOX, for measurable liver metastases between 1 January 2017 and 31 June 2025, were identified from Nanfang Hospital and Zhujiang Hospital. Since bevacizumab serves as a first-line treatment for RAS-mutant cases but demonstrates suboptimal efficacy in B-Raf Proto-Oncogene (BRAF)-mutant cases [12], only patients with confirmed RAS mutations were enrolled in this study. Detailed information on the standard chemotherapy regimens is available in Supplementary Material S2; detailed information regarding the patients and treatment regimens is available in Tables S3a,b and S4. This registry prospectively collects anonymized clinical, genomic, and imaging data, with independent auditing ensuring >95% data completeness and validity.

### 2.2. Inclusion and Exclusion Criteria

Inclusion criteria: [1] Patients with colorectal adenocarcinoma and liver metastasis confirmed by CECT, meeting the following requirements: RAS mutations confirmed by genetic testing; having received at least 3 cycles of bevacizumab-containing treatment; and chemotherapy regimens limited to standard ones (including FOLFOX, FOLFIRI, or CAPEOX). Details of chemotherapy regimens for each patient are provided in Table S4 [2]. Availability of dual-phase CECT (arterial, portal venous) performed ≤4 weeks before bevacizumab initiation [3]. Radiologically evaluable liver metastases (≥1 lesion, ≥4.5 mm) [4]. No metastatic sites other than liver metastases [5]. Documented treatment response status (resistant vs. responsive).

Exclusion criteria: [1] Prior liver-directed therapy (e.g., ablation) [2]. Patients who do not have RAS mutations [3]. Use of chemotherapy regimens other than the standard FOLFOX, FOLFIRI, or CAPEOX in combination with bevacizumab [4]. Incomplete CT imaging protocols (missing phases) [5]. Images acquired with non-standardized acquisition parameters (e.g., inconsistent scanner models or tube voltage) to ensure data homogeneity [6]. Severe motion artifacts or significant anatomical misregistration between the arterial phase (AP) and portal venous phase (PVP) that could not be corrected manually.

### 2.3. Ethical Considerations

The study protocol was reviewed and approved by the Ethics Committee of ZhuJiang Hospital, Southern Medical University (Approval No. 2024KY18101), and the Human Subjects Ethics Subcommittee of The Hong Kong Polytechnic University (Approval No. HSEARS20250812004). As the study involved the prospective collection of anonymized data, the ethics committees determined that informed consent was not required.

### 2.4. Outcomes and Definitions

Outcome: Bevacizumab-based chemotherapy resistance was defined as radiological progression of target liver metastases during first-line bevacizumab-containing therapy. Progression was determined by the following:

(1) ≥20% increase in the sum of target lesion diameters (minimum 5 mm absolute increase).
(2) Appearance of ≥1 new metastatic lesion.

All imaging biomarkers were evaluated by two independent radiologists blinded to clinical outcomes (κ > 0.80 for all critical features). Discordant cases underwent consensus review with a third senior radiologist. Furthermore, the inter-observer agreement for 2D ROI delineation was assessed, yielding a mean Dice Similarity Coefficient (DSC) of 0.917 ± 0.044 for the AP and 0.926 ± 0.026 for the PVP, as provided in Table S1. In addition, each radiologist ensured that the liver images of the dual phase were in the same plane, and images that did not meet this condition were excluded from the study. In the two-pane split-screen comparison window of the image reading software, the PVP was selected as the reference. The target plane where the maximum cross-section of the tumor was located was identified, and the images of the AP were fine-tuned using the “manual slice adjustment function” of the image reading software with the target anatomical plane of the PVP as the standard so as to ensure that both phases in the same image were in the same plane. To ensure transparency regarding image quality, detailed descriptions of the acquisition protocols (including scanner models, tube voltage, and current settings) are provided in Supplementary Material S1.

Key variables:

(1) Tumor response status.
(2) Resistant: Meet the criteria for progression and stability.
(3) Responsive: Partial/complete response (≥30% decrease in target lesions).

Treatment duration: Time from bevacizumab initiation to progression (resistant) or last effective cycle (responsive).

### 2.5. CT Labeling

The pipeline used Bee DICOM Viewer V3.7.2 (https://beedicom.com) for the image processing part (Bee DICOM Viewer is open-source research software and is not FDA approved for real case PSI AM).

The image processing evaluated in this study was performed on the same hardware in a systematic procedure on a laptop with an Intel Core i7 (Intel, Santa Clara, CA, USA). Each user performed the segmentations on this hardware with the same version of Bee DICOM Viewer.

The user first marked the liver metastasis regions of the image of dual-phase CECT manually. In accordance with standardized oncologic imaging protocols, physicians selected the axial dual-phase CECT slice exhibiting the maximal cross-sectional area of each target lesion for contour delineation. This approach aligns with RECIST 1.1 guidelines, which mandate measurement of the longest axial diameter within the plane of maximal tumor dimension to ensure reproducibility and comparability across serial assessments. And then extracted features 2 mm outward and 2 mm inward from the edge of each liver metastasis lesion, as well as in the center of the tumor.

### 2.6. Image Preprocessing and Radiomic Features Extraction

Image processing and radiomic feature extraction were performed using a Python-based pipeline incorporating SimpleITK (version 2.5.0), OpenCV (version 4.11.0), and PyRadiomics (version 3.1.0) libraries. To comprehensively capture lesion characteristics, our analysis utilized four distinct data inputs: original AP and PVP images and two derived datasets—AP-PVP subtraction images and DeltaP features.

The dual phase (AP, PVP) is designed to maximize informational content for model training. The conventional reliance on the PVP in CRLM radiomics [19] discards potentially complementary data from the AP. Though prior work often favored PVP in CRLM radiomics analyses [20], we contend that the two phases contain synergistic information. This is empirically supported by an ablation study on CRLM prediction, which showed that while a PVP-only model (AUC = 0.752) outperformed an AP-only model (AUC = 0.653), the performance of either single-phase model was significantly lower than that of the combined two-phase approach, leading to the conclusion that “a multi-phase approach is more useful” [21]. Inspired by this data-driven evidence, our study leverages both phases to not only improve predictive accuracy but also to explicitly quantify the dynamic changes between them.

The AP-PVP subtraction images were generated via pixel-wise intensity subtraction to show differential contrast enhancement [22]. DeltaP radiomic features were calculated by dividing the feature values extracted from AP images by their corresponding values from PVP images [23]. Images and corresponding masks were resampled to an isotropic pixel size of 1.0 × 1.0 mm to ensure spatial standardization [24]. Image intensities were subsequently discretized using a fixed bin width of 25 Hounsfield Units to normalize intensity resolution across all patients [25].

**Figure 1:**
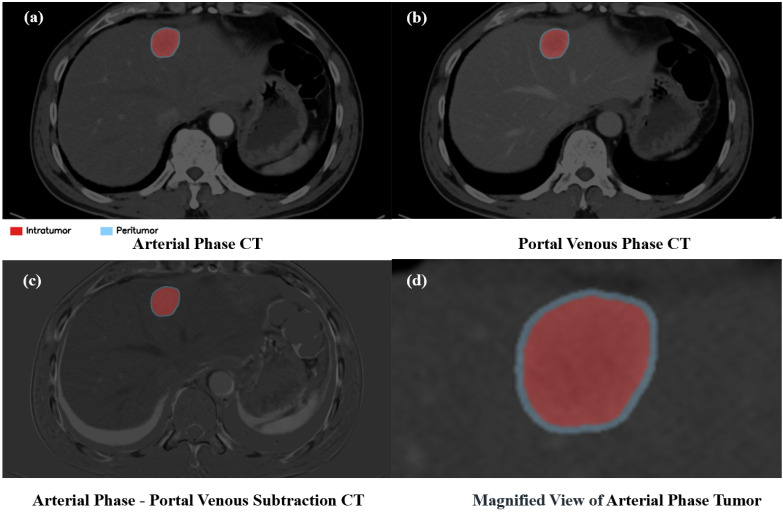
Segmentation of the ROIs for radiomic feature extraction. (**a**) AP image with the segmented core tumor (red) and peritumor (blue) ROIs. (**b**) The same lesion and ROIs shown in the PVP image. (**c**) The corresponding AP-PVP subtraction images, generated to highlight contrast enhancement dynamics. (**d**) A magnified view of the AP ROIs, clearly delineating the boundary between the two segmented regions.

Radiomic features were extracted from three distinct regions of interest (ROIs): (i) the primary tumor contour, manually delineated by an experienced physician; (ii) a 2 mm intratumoral core, generated via a 2 mm erosion of the tumor boundary; and (iii) a 4 mm peritumoral ring, constructed as the volumetric shell between a 2 mm dilation and the 2 mm erosion of the primary contour [23]. These morphological operations were performed on the resampled images to ensure the distances were in precise physical units of millimeters. A demonstrative example of the intratumoral and peritumoral region is shown in Figure 1.

For each ROI, 2D radiomic features were computed from three distinct image sets: non-derived AP and PVP images and a derived AP-PVP subtraction image. To capture multi-scale textural information, a comprehensive feature extraction pipeline was applied to each set. This process involved analyzing seven images per ROI: the original unfiltered image and six filtered images derived from it using three Laplacian of Gaussian (LoG) filters (σ = 1.0, 2.0, 3.0) and three wavelet-based filters (db1, db2, sym2) [22,26]. From each of these seven images, a baseline set of 102 feature classes was extracted, comprising shape (*n* = 9), first-order statistics (*n* = 18), and second-order texture (*n* = 75). The second-order texture features were calculated from five matrices: gray-level co-occurrence (GLCM; *n* = 24), gray-level run-length (GLRLM; *n* = 16), gray-level size zone (GLSZM; *n* = 16), gray-level dependence (GLDM; *n* = 14), and neighboring gray tone difference (NGTDM; *n* = 5).

A detailed breakdown of the feature calculation process, which is distinguished between non-derived and derived phases, is summarized in Table 1. For the non-derived AP and PVP, applying the 102 feature classes to all seven images generated 714 features per ROI, totaling 2142 features per phase. In contrast, for the derived phases (e.g., AP-PVP subtraction, DeltaP), the nine shape2D features were excluded in accordance with PyRadiomics guidelines [27]. This modified approach yielded 651 features per ROI from the remaining 93 non-shape classes, totaling 1953 features per derived phase. All radiomic features were defined and calculated in strict accordance with the standards set by the Image Biomarker Standardization Initiative (IBSI) [28], providing a reproducible foundation for future validation studies.

### 2.7. Feature Analysis

The feature analysis was based on a total cohort of 42 patients, who contributed 73 analyzable samples. To ensure strict independence between model development and validation, this cohort was stratified based on patient admission time into a discovery cohort (30 patients, 51 samples) and an independent validation cohort (12 patients, 22 samples). Crucially, to prevent data leakage and ensure unbiased model development, all subsequent feature analysis and selection procedures described in this section were conducted exclusively on the discovery cohort. Thus, the patient data involved in the model validation are not involved in any of the feature analyses and model development in the discovery cohort, thus safeguarding independent model validation.

To identify a non-redundant and highly predictive feature, a four-stage selection pipeline was designed and executed independently for each of the four feature groups in the train cohort (i.e., AP, PVP, AP-PVP, and DeltaP). First, a two-sided independent Mann–Whitney U test was applied to all radiomic features to identify those significantly associated with treatment response, retaining only features with a *p*-value < 0.05 for subsequent analysis. Second, to reduce feature redundancy, a clustering-based feature selection approach was employed on the features retained from the previous step [29]. Specifically, these features were grouped using the K-Means clustering algorithm. To mitigate bias from initial centroid placement, the K-Means algorithm was run 100 times with different random initializations, and the clustering result with the lowest inertia was selected [23]. Third, a single representative feature was selected from each cluster. The feature with the lowest *p*-value from the initial Mann–Whitney U test was chosen as the representative for its respective cluster. The number of clusters (k) was individually tuned for each phase to optimize the univariate predictive performance of the resulting feature set. The selection of the best k was evaluated in the following order of priority:

(1) Maximization of the average Area Under the Curve (AUC) evaluated in the discovery cohort.
(2) Stability of the confidence intervals (95% CIs).
(3) Model parsimony, the principle of selecting the simplest model (i.e., with the minimum number of features) among those with comparable performance [30].

Finally, the *p*-values of the candidate features selected from the previous stage were adjusted for multiple comparisons using the Benjamini–Hochberg (BH) procedure to control the false discovery rate (FDR). Only features with an FDR-adjusted *p*-value (q-value) < 0.1 were retained as the final predictive radiomic feature set for subsequent model development.

To assess the individual predictive performance of each feature in the final radiomic feature set, Logistic Regression analysis was applied; the area under the receiver operator characteristic (ROC) curve (AUC), sensitivity (Sen), and specificity (Spe) were then reported. Prior to regression analysis, all features were rescaled to a mean of 0 and a standard deviation of 1. All statistical analyses were implemented in Python (version 3.9.22) [23].

### 2.8. Radiomic Model Development and Evaluation

The entire feature selection process described below was conducted exclusively on the training cohort. The process began with the set of statistically significant features identified from the statistical analysis (FDR < 0.1), as described in Section 2.7. From this pre-filtered feature dataset, a final predictive feature set was selected using the Least Absolute Shrinkage and Selection Operator (LASSO) regression. By applying an L1 penalty, LASSO performs embedded feature selection by shrinking the coefficients of non-informative features to exactly zero [31]. This entire selection protocol was executed independently for each of the four feature categories (i.e., AP, PVP, AP-PVP, and DeltaP). To construct the multi-phasic model, the FDR-filtered features from all four feature categories were first consolidated into a single comprehensive set, to which the same rigorous LASSO cross-validation procedure was then applied to derive a synergistic feature set. Subsequently, to minimize multicollinearity and eliminate redundancy, Spearman’s rank correlation analysis was performed on the LASSO-selected features. Features exhibiting a high correlation coefficient (|ρ| > 0.7) were iteratively removed to yield the final predictive feature set [32].

To predict treatment response, six machine learning models were constructed: Logistic Regression (LR), Decision Tree (DT), Random Forest (RF), Support Vector Machine (SVM), K-Nearest Neighbors (KNNs), and Naïve Bayes (NB) [33]. Prior to model training, all features were subsequently standardized to have a mean of 0 and a standard deviation of 1. To control variability during model training, the random state parameter was also set to 42 for all applicable models (LR, DT, RF, and SVM). The other benchmarked models (KNNs and NB) are deterministic in their standard implementation and do not require a random seed.

Model hyperparameter optimization was performed on the training cohort using a 3-fold stratified cross-validation scheme integrated within a grid search (GridSearchCV). The performance of the optimized model on the training data was then estimated by calculating the mean and standard deviation of the AUC.

Following hyperparameter selection, each classifier was retrained on the entire training cohort using its identified optimal parameters. The final generalization performance of the models was then evaluated on the independent test cohort. Performance was assessed using the AUC, Sen, and Spe. The optimal classification threshold used to calculate Sen and Spe for the test set was determined on the training cohort by maximizing Youden’s J statistic [34]. To elucidate the decision-making process of the optimal predictive model, we employed Shapley Additive Explanations (SHAP), and to investigate the distributional characteristics of the features comprising the final radiomic feature set, we employed violin plots. To further evaluate the clinical utility of the final model in Table S2, we performed several additional analyses as per the TRIPOD reporting guidelines.

## 3. Results

### 3.1. Patient Characteristic

Table 2 summarizes patient characteristics. This study included 42 patients (25 male/17 female) with a mean age of 55.90 ± 10.88 years. Bevacizumab was administered at dosages of 200 mg (*n* = 3), 300 mg (*n* = 20), 400 mg (*n* = 16), 500 mg (*n* = 2), and 600 mg (n = 1). Dosing intervals were 2 weeks (*n* = 16), 3 weeks (*n* = 25), and 4 weeks (*n* = 1). The average tumor nodule diameter was 6.46 ± 5.84 cm.

A total of 42 patients were included in this study, contributing a total of 73 samples for analysis. To ensure strict independence between model development and validation, the cohort was partitioned at the patient level.

The training set comprised 30 patients, who provided 51 samples. Within these samples, 27 (52.9%) were from responder patients, and 24 (47.1%) were from non-responder patients. The independent validation set consisted of 12 patients, providing the remaining 22 samples. Of these, 10 (45.5%) were from responder patients and 12 (54.5%) were from non-responder patients. This patient-level split guarantees that no data from a single patient appeared in both the training and validation sets.

### 3.2. Clinical Associations Between Features and Treatment Response

To determine the optimal number of clusters for the K-Means algorithm, hyperparameter tuning was performed on the training cohort. Based on Section 2.7, an optimal cluster count of k = 3 was identified for each of the four feature groups (AP, PVP, AP-PVP, and DeltaP).

Table 3 summarizes the final list of 12 candidate predictors identified as significantly associated with treatment response (FDR-adjusted *p*-value < 0.1, ranging from 0.005 to 0.029) for AP, PVP, AP-PVP subtraction, and DeltaP groups. The peritumoral region was the major source of radiomic predictors (n = 8/12, 67%), in stark contrast to the whole tumor (n = 3/12, 25%) and intratumoral regions (n = 1/12, 8%). Furthermore, features generated from LoG-filtered images were highly prevalent (n = 8/12, 67%), suggesting the importance of accentuated textural patterns in predicting treatment response.

Among the imaging phases, AP radiomic features accounted for the predictors with the highest statistical significance. All three AP predictors were peritumoral LoG-filtered textural features (GLCM_Maximum Probability, GLCM_Inverse Variance, GLSZM_Zone Variance), demonstrating the lowest FDR values (FDR = 0.005) and yielding AUC between 0.719 and 0.755. This was followed by features from the DeltaP phase, where all three predictors were also highly significant with a uniform FDR of 0.009 (GLRLM_Run Length Non-Uniformity, GLDM_Dependence Non-Uniformity, GLDM_Small Dependence Emphasis), yielding AUC between 0.713 and 0.738. Significant contributions were also provided by the PVP and AP-PVP, yielding AUC between 0.679 and 0.721. However, it is worth noting that no 2D shape-based features were found to be significantly different between the responder and non-responder groups.

### 3.3. Performance of Single-Phase and Derived-Phase Models

Table 4 summarizes the comparative performance of six machine learning classifiers developed for each of the four single-phase radiomic feature sets. Models were assessed using a 3-fold cross-validation on the training set and subsequently validated on the independent testing set.

Among the single-phase models, the PVP radiomic feature set yielded the most promising performance, with a KNN classifier achieving an AUC of 0.671 on the testing set. In contrast, models from other phases showed limited predictive utility. The optimal models for the AP, AP-PVP subtraction, and DeltaP sets yielded AUCs of 0.583 (LR/SVM), 0.600 (DT), and 0.650 (NB) on the testing set, respectively.

While these results underscore the value of individual imaging phases, particularly PVP, they also suggest that reliance on a single imaging moment may render suboptimal model performance. The presence of predictive signals across multiple phases indicated that a more robust and comprehensive model could be engineered by integrating these complementary data sources.

### 3.4. Performance of the Combined Multi-Phasic Model

To test the hypothesis that an integrated model could outperform single-phase approaches, a combined multi-phasic radiomic feature set was constructed following the methodology detailed in Section 2.8. As presented in Table 5, the NB classifier emerged as the best-performing model, achieving an AUC of 0.717, a Sen of 0.900, and a Spe of 0.500 on the independent test set. This indicates that while the model is highly effective at identifying patients who are likely to respond to bevacizumab-based chemotherapy (i.e., a low false-negative rate), it concurrently tends to misclassify a significant portion of non-responders as responders (i.e., a high false-positive rate).

The NB classifier (testing AUC: 0.717) outperformed the best single-phase model (testing AUC: 0.671), confirming the added value of integrating multi-phasic information for predicting treatment response. Consequently, this optimal NB-based radiomic feature set was carried forward for in-depth interpretability analysis.

### 3.5. Interpretation of the Optimal Radiomic Features

**Figure 2:**
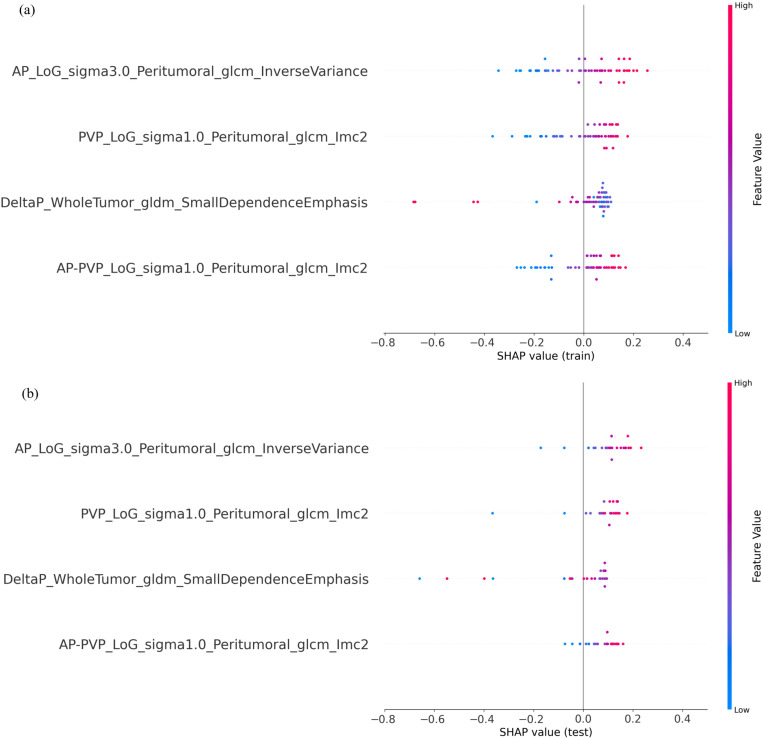
SHAP summary plots validating the interpretability and robustness of the optimal combined NB model. (**a**) Analysis of the training cohort (n = 51). (**b**) Analysis on the independent test cohort (n = 22). In both plots, each point represents a sample. The *y*-axis shows feature importance, and the *x*-axis shows the feature’s impact on model output (positive SHAP values drive the prediction toward “Responder”). Color indicates the feature’s value (blue = low, red = high).

To provide insight into the model’s decision-making process, the final multi-phasic NB model was interpreted using SHAP. The SHAP summary plots revealed that the four radiomic features influenced the model’s prediction through two distinct trends in Figure 2a,b. The first trend, a negative correlation, was exemplified by the one influential predictor: GLDM Small Dependence Emphasis (from the Delta radiomics feature), where low feature values drove the model’s prediction toward the responder group. In contrast, the opposite trend of positive correlation was exhibited by the other three features: GLCM Inverse Variance (from AP images) and the two GLCM Informational Measure of Correlation 2 features (from AP-PVP subtraction and PVP images, respectively), where high values drove the model’s prediction toward the responder group. The consistent manifestation of these two opposing trends across both training and test cohorts in the SHAP analysis demonstrates the stability of the model’s internal logic. Feature correlation analysis confirms that the selected multi-phasic features provide complementary information with controlled redundancy in Figure S1.

**Figure 3:**
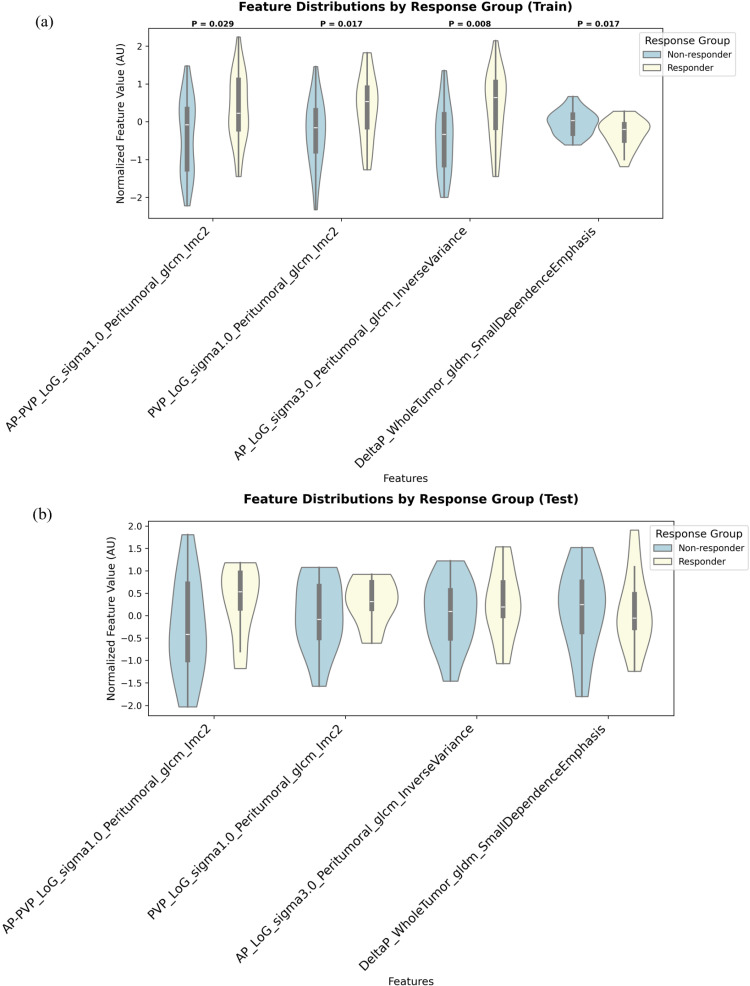
Violin plots showing the distribution of the four radiomic features in the final feature set, stratified by treatment response. (**a**) Distributions in the training cohort (n = 51). (**b**) Distributions in the independent test cohort (n = 22). Each violin illustrates the probability density of the normalized feature values for the non-responder (blue) and responder (yellow) groups. The inner box plot shows the median and interquartile range. *p*-values from the Mann–Whitney U test are displayed for the training cohort, showing significant differences between groups for all features.

The violin plots providing visual corroboration of the SHAP analysis findings are shown in Figure 3a,b. A Mann–Whitney U test confirmed that the distributional differences between the responder and non-responder groups were statistically significant for all four features in the training cohort (all *p* < 0.05, Figure 3a). For the one radiomic feature with a negative correlation, the responder group exhibited lower values than the non-responder group. Conversely, for the three radiomic features with a positive correlation, the responder group indeed exhibited higher values. This visual and statistical consistency across both the training and independent test cohorts provides evidence for the robustness of the identified predictive features.

## 4. Discussion

For patients with RAS-mutated CRLMs, selecting which patients will benefit from bevacizumab-based chemotherapy is a critical clinical challenge, aiming to maximize efficacy while sparing non-responders from ineffective therapy. This need has driven the development of powerful predictive models, which increasingly employ complex ‘black box’ architectures such as deep learning. For example, Zhou et al. developed a multicenter deep radiomics fusion model integrating baseline PET/CT, clinical variables, and biopsy data to predict bevacizumab response in CRLMs [35], and similarly, an MRI-based model integrated radiomics, deep learning, and clinical features, achieving efficient CRLM prediction with SHAP interpretation [36]. Although these studies provide post hoc explanations (e.g., heat maps or SHAP values) to indicate ROI, their limited intrinsic interpretability hinders the understanding of which biologically meaningful image attributes drive prediction. Distinct from these approaches, the primary advantage of our study is model interpretability design, achieved through the integration of multi-phasic dynamics (i.e., AP, PVP, AP-PVP, and DeltaP) with spatial Spe (i.e., peritumor, core tumor, and whole-tumor regions). We developed and validated a multi-phasic CT radiomic-based model to investigate the interpretability of the identified radiomic features to elucidate the potential biological characteristics underlying treatment response.

A consistent quantitative signal from our analysis localizes the primary source of predictive information to the peritumoral region. During feature selection, a majority of initial candidate predictors (8/12, 67%) and an even larger share of the final feature set (3/4, 75%) were derived from peritumoral ROIs. This spatial localization is biologically plausible because bevacizumab neutralizes secreted VEGF-A, thereby modulating angiogenesis specifically at the tumor-host interface [37]; therefore, our imaging features tend to capture downstream correlations of that biology. Indeed, the peritumoral zone is widely recognized as a focus of angiogenic and invasion-related activity. Analogous studies in hepatocellular carcinoma (HCC) have shown that margin radiomic features predict microvascular invasion (MVI), a histologic marker of aggressive angiogenesis [22,38]. More broadly, the idea that the tumor-stroma boundary harbors critical imaging biomarkers has been validated across malignancies, including breast, head and neck, lung, and gastric cancers [39,40,41,42]. Our results are consistent with these findings and suggest that peritumoral information is similarly important for predicting response to vascular-targeted therapy in RAS-mutated CRLMs.

Beyond spatial localization, biomarkers of textural heterogeneity and complexity are central determinants of predicted bevacizumab response. In our study, the final feature set consists exclusively of advanced textural features, specifically those accentuated by LoG filtering. This aligns with radiomic best practices and reviews, indicating that texture measures capture subtle biological heterogeneity beyond morphology [43]. SHAP analysis identified a LoG-filtered AP peritumoral GLCM_Inverse Variance. Notably, the GLCM can quantify textural information [44], and radiogenomic studies have associated similar GLCM metrics with KRAS mutation status in CRLMs [45]. Together, these findings suggest that CT texture is unlikely to be random noise and may act as a reproducible, non-invasive surrogate for aspects of genomic instability and microstructural disorder that affect Sen to anti-angiogenic therapy.

The model’s apparent robustness arises from synergistic integration across spatial and temporal domains rather than reliance on any single metric. Our findings align with the evolving landscape of precision oncology in CRLMs. While recent studies have validated the use of post-treatment radiomics to estimate pathologic response [46] or pretreatment quantitative analysis to predict volumetric reduction [47], our study specifically highlights the incremental value of multi-phasic and peritumoral features prior to bevacizumab administration. Multi-phasic imaging often outperforms single-phase approaches in liver malignancies [48], and our model aligns with this principle. It also incorporates temporal dynamics via a whole tumor delta radiomic metric (GLDM_Small Dependence Emphasis), consistent with studies utilizing delta radiomics to quantify biological shifts across enhancement phases [49]. Two peritumoral Informational Measure of Correlation 2 (Imc2) features from PVP and AP-PVP subtraction images capture irregular wash-in/wash-out dynamics. Such unpredictable enhancement patterns plausibly reflect the inefficient, chaotic vascular network.

Viewed mechanistically, these observations support the interpretation that our multi-phasic model non-invasively profiles dimensions of tumor angiogenesis: peritumoral features may mark the spatial footprint of vascular influence, dynamic multiphase measures quantify aberrant perfusion, and texture encodes the resulting chaotic microarchitecture. Framed against the hallmarks of cancer, the model appears to capture aspects of the “inducing angiogenesis” hallmark [50], which provides a plausible rationale for its association with bevacizumab response, although this remains a hypothesis until paired histologic or molecular validation is performed.

From a potential clinical-use perspective, if validated, our radiomics model, trained using pre-therapeutic multi-phasic CT images, serves as a non-invasive biomarker to help stratify patients for bevacizumab therapy prior to treatment administration. The model demonstrated high Sen (90%) at the expense of low Spe (50%). This high Sen is advantageous as a screening tool to minimize missed responders, ensuring that patients likely to benefit are not deprived of potentially life-extending therapy. However, modest Spe implies a risk of overestimating the responder group. Therefore, to better exclude non-responders and avoid unnecessary toxicity, pragmatic deployment would likely require combining these radiomic features with established molecular markers (e.g., RAS/BRAF status) or other clinical variables, rather than using the model as the sole determinant of therapy.

This study has several limitations. First, as a pilot investigation focuses on a specific molecular subgroup (RAS-mutated), the sample size is limited (n = 42), which subjects the results to potential selection bias. Consequently, the results of this pilot study should be interpreted cautiously. Second, while our 2D ROIs were drawn from the maximal tumor cross-section to ensure reproducibility, this approach may not fully capture the complete 3D heterogeneity of the tumor and its microenvironment. And the criterion of measurable lesions was set at ≥4.5 mm. The implications of this threshold selection are that our model might be less sensitive to very small metastatic deposits. Third, this study lacks a “chemotherapy-only” control group. Consequently, we cannot definitively disentangle whether the identified radiomic features are specifically predictive of bevacizumab benefit or prognostic for general chemotherapy sensitivity. Fourth, although our mechanistic interpretations are biologically plausible and consistent with the current body of literature, this cohort lacked paired histological or genomic data to directly validate the proposed biology links. Finally, the radiomic features were extracted from images strictly selected based on standardized acquisition protocols. While this minimized technical variability for model training, the generalizability of our findings to images acquired with non-standardized parameters or different scanner vendors remains validated in broader populations.

Several avenues for future research are critical for its clinical translation. First and foremost, prospective, multicenter validation is imperative to confirm the generalizability of our peritumoral-based findings across diverse patient populations and imaging protocols [51]. Second, to further validate the specificity of our identified features, future studies will aim to include a control cohort (receiving chemotherapy without bevacizumab) to definitively distinguish bevacizumab-specific predictive effects from general prognostic factors. Third, we plan to implement 3D volumetric segmentation. This will allow for a more comprehensive assessment of tumor heterogeneity and verify whether volumetric features offer incremental predictive value over the robust 2D markers identified in this study. Fourth, to elucidate the biological underpinnings of our peritumoral imaging phenotype, radiogenomic analysis is a vital next step. Future work should aim to correlate the significant peritumoral features we identified with underlying molecular data (e.g., RAS status) or transcriptomic profiles related to angiogenesis and immune infiltration [52,53]. Finally, future work will leverage deep learning techniques in two key areas: exploring hybrid models that combine radiomic features with deep representations to enhance predictive power and developing fully automated segmentation pipelines (e.g., UNet/C-Enet) to streamline clinical implementation [54].

## 5. Conclusions

In this pilot study, our preliminary results suggest that a combined multi-phasic radiomic feature model, driven primarily by peritumoral features from arterial, portal venous, and derived data, including AP-PVP subtraction and DeltaP radiomics (characterizing textural heterogeneity), was associated with response to bevacizumab-based chemotherapy in patients with RAS-mutated CRLMs. A notable observation was that peritumor LoG-filtered radiomic features demonstrated higher predictive potential compared to core tumor features. The optimal combined-phase model, NB algorithm, achieved an encouraging predictive performance (Avg. training AUC: 0.731, Avg. testing AUC: 0.717). These findings highlight the potential of our interpretable model for the pre-treatment stratification of patients for bevacizumab-based chemotherapy. Given the limited sample size, a larger prospective cohort is essential to validate these initial observations and confirm their clinical utility.

## Figures and Tables

**Table 1:** Summary of the number of radiomic features extracted per ROI for each phase type. ^1^ Non-derived Phases (AP, PVP): Full feature set (102 classes) applied to 7 image sets (1 original + 6 filtered). ^2^ Derived Phases (AP-PVP subtraction, DeltaP): Reduced feature set (93 classes, excluding 2D shape features) applied to 7 image sets.

# of Features	Peritumoral ROI	Tumor Core ROI	Whole Tumor ROI
Non-derived Phases ^1^	714	714	714
Derived Phases ^2^	651	651	651

Note: # indicates number.

**Table 2:** Patient characteristics.

Total Number of Patients	42
Gender	
• Male	25
• Female	17
Age (y), mean ± SD	55.90 ± 10.88
• 30–39	2
• 40–49	12
• 50–59	13
• 60–69	10
• 70–79	5
Sum of diameter of tumor nodule (cm), mean + SD	6.46 ± 5.84
Resistance to Bevacizumab-based Chemotherapy?	
• Yes	21
• No	21
Bevacizumab Dosage (mg)	
• 200	3
• 300	20
• 400	16
• 500	2
• 600	1
Dosing Interval (week)	
• 2	16
• 3	25
• 4	1

**Table 3:** A list of the 12 independent radiomic predictors that demonstrated a significant association with response to bevacizumab-based chemotherapy. Statistical significance is indicated by the false discovery rate (FDR)-adjusted *p*-value after applying the Benjamini–Hochberg (BH) procedure for multiple test corrections. AUC, Sen, Spe, and *p*-value obtained from LR for each of these independent significant predictors are also reported.

Features	FDR Adjusted *p*-Value	AUC	Sen	Spe
***AP radiomic features*** *Second-order textural features*				
GLCM_Maximum Probability (LoG, σ = 3, Peritumoral)	0.005	0.744 (0.600–0.871)	0.741	0.708
GLCM_Inverse Variance (LoG, σ = 3, Peritumoral)	0.005	0.755 (0.609–0.879)	0.593	0.875
GLSZM_Zone Variance (LoG, σ = 2, Peritumoral)	0.008	0.719 (0.573–0.860)	0.704	0.708
***PVP radiomic features*** *First-order statistical features*				
First-order_10th Percentile (LoG, σ = 2, Peritumoral)	0.013	0.713 (0.557–0.848)	0.444	0.917
*Second-order textural features*				
GLCM_Informational Measure of Correlation 2 (LoG, σ = 1, Peritumoral)	0.013	0.721 (0.566–0.853)	0.741	0.625
NGTDM_Complexity (LoG, σ = 3, Intratumoral)	0.028	0.681 (0.494–0.830)	0.926	0.542
***AP-PVP Subtraction radiomic features*** *First-order statistical features*				
First-order_Skewness (Unfiltered, Whole Tumor)	0.021	0.721 (0.567–0.852)	0.852	0.667
*Second-order textural features*				
GLCM_Inverse Variance (LoG, σ = 3, Peritumoral)	0.021	0.702 (0.548–0.836)	0.815	0.583
GLSZM_Zone Variance (LoG, σ = 2, Peritumoral)	0.029	0.679 (0.520–0.827)	0.704	0.667
***DeltaP radiomic features*** *Second-order textural features*				
GLRLM_Run Length Non-Uniformity (Unfiltered, Whole Tumor)	0.009	0.713 (0.568–0.850)	0.593	0.792
GLDM_Dependence Non-Uniformity (Wavelet, db1, Peritumoral)	0.009	0.721 (0.561–0.855)	0.815	0.542
GLDM_Small Dependence Emphasis (Unfiltered, Whole Tumor)	0.009	0.738 (0.589–0.870)	0.778	0.667

**Table 4:** Comparative performance of six machine learning classifiers across the combined multi-phase model, evaluated on the training set (3-fold cross-validation) and the independent test set.

Feature Set	Classifier	Train AUC (Mean ± Std)	Test AUC (95% CI)	Test Balanced Accuracy	Sen	Spe
AP	LR	0.755 ± 0.069	0.583 (0.321–0.838)	0.608	0.800	0.417
	DT	0.713 ± 0.043	0.546 (0.309–0.767)	0.575	0.900	0.250
	RF	0.764 ± 0.065	0.479 (0.222–0.733)	0.483	0.800	0.167
	SVM	0.755 ± 0.069	0.583 (0.330–0.831)	0.608	0.800	0.417
	KNN	0.722 ± 0.087	0.562 (0.300–0.797)	0.442	0.800	0.083
	NB	0.731 ± 0.040	0.567 (0.306–0.810)	0.608	0.800	0.417
PVP	LR	0.727 ± 0.094	0.617 (0.330–0.854)	0.583	1.000	0.167
	DT	0.544 ± 0.122	0.517 (0.325–0.722)	0.517	0.700	0.333
	RF	0.514 ± 0.152	0.621 (0.376–0.859)	0.608	0.800	0.417
	SVM	0.727 ± 0.094	0.383 (0.140–0.629)	0.500	0.000	1.000
	KNN	0.537 ± 0.047	0.671 (0.471–0.857)	0.658	0.900	0.417
	NB	0.713 ± 0.082	0.617 (0.375–0.842)	0.583	1.000	0.167
AP-PVP Subtraction	LR	0.759 ± 0.200	0.492 (0.242–0.743)	0.400	0.800	0.000
	DT	0.597 ± 0.049	0.600 (0.362–0.813)	0.542	0.500	0.583
	RF	0.623 ± 0.101	0.337 (0.090–0.603)	0.483	0.800	0.167
	SVM	0.759 ± 0.200	0.508 (0.225–0.760)	0.400	0.800	0.000
	KNN	0.634 ± 0.118	0.367 (0.141–0.616)	0.467	0.600	0.333
	NB	0.741 ± 0.204	0.417 (0.170–0.686)	0.400	0.800	0.000
DeltaP	LR	0.750 ± 0.090	0.508 (0.256–0.775)	0.517	0.700	0.333
	DT	0.609 ± 0.190	0.450 (0.201–0.714)	0.500	0.500	0.500
	RF	0.676 ± 0.198	0.446 (0.196–0.700)	0.425	0.600	0.250
	SVM	0.750 ± 0.090	0.492 (0.225–0.723)	0.500	0.000	1.000
	KNN	0.697 ± 0.126	0.396 (0.161–0.620)	0.458	0.500	0.417
	NB	0.565 ± 0.076	0.650 (0.396–0.876)	0.592	0.600	0.583

**Table 5:** Performance of machine learning classifiers using the combined multi-phasic LASSO-selected feature set. The best-performing model is highlighted in bold.

Feature Set	Classifier	Train AUC (Mean ± Std)	Test AUC (95% CI)	Test Balanced Accuracy	Sen	Spe
Combined	LR	0.847 ± 0.120	0.608 (0.366–0.857)	0.583	1.000	0.167
	DT	0.685 ± 0.135	0.550 (0.308–0.763)	0.517	0.700	0.333
	RF	0.801 ± 0.105	0.592 (0.325–0.842)	0.625	1.000	0.250
	SVM	0.843 ± 0.118	0.583 (0.325–0.846)	0.583	1.000	0.167
	KNN	0.787 ± 0.114	0.579 (0.348–0.830)	0.617	0.900	0.333
	NB	0.731 ± 0.098	0.717 (0.473–0.929)	0.700	0.900	0.500

## Data Availability

The data presented in this study are available on request from the corresponding author. The data are not publicly available due to patient privacy protection.

## Supplementary Materials

The following supporting information can be downloaded at: https://www.mdpi.com/article/10.3390/bioengineering13020137/s1, Supplementary Material S1. Acquisition Equipment; Supplementary Material S2. Detailed Information on Treatment Regimens; Figure S1: Correlation Matrix of the Final Radiomic Features; Table S1: Inter-observer agreement (Dice Similarity Coefficient) of 2D tumor ROI delineation between 2 radiologists in Arterial Phase (AP) and Portal Venous Phase (PVP); Table S2. TRIPOD Checklist for Prediction Model Development and Validation; Table S3a. Sum of diameter of tumor nodule in Train Cohort; Table S3b. Sum of diameter of tumor nodule in Test Cohort; Table S4. Patient Characteristic Information.

## Abbreviations

AP	Arterial phase
AUC	Area Under the Curve
BH	Benjamini–Hochberg
BRAF	B-Raf Proto-Oncogene
CI	Confidence interval
CECT	Contrast-enhanced computed tomography
CRC	Colorectal cancer
CRLMs	Colorectal liver metastases
CT	Computed tomography
DSC	Dice Similarity Coefficient
DT	Decision Tree
DeltaP	Delta phase
FDG-PET	Fluorodeoxyglucose positron emission tomography
FDR	False discovery rate
GLCM	Gray-level co-occurrence matrix
GLDM	Gray-level dependence matrix
GLRLM	Gray-level run-length matrix
GLSZM	Gray-level size zone matrix
HCC	Hepatocellular carcinoma
IBSI	Image Biomarker Standardization Initiative
KRAS	Kirsten rat sarcoma virus
KNN	K-Nearest Neighbor
LoG	Laplacian of Gaussian
LASSO	Least Absolute Shrinkage and Selection Operator
LR	Logistic Regression
LTB	Low tumor burden
MRI	Magnetic resonance imaging
mCRC	Metastatic colorectal cancer
MVI	Microvascular invasion
NB	Naïve Bayes
NGTDM	Neighboring Gray Tone Difference Matrix
NRAS	Neuroblastoma RAS viral oncogene homolog
OS	Overall survival
PVP	Portal venous phase
RAS	Rat sarcoma virus
RF	Random Forest
ROC	Receiver operator characteristic
ROI	Region of Interest
Sen	Sensitivity
Spe	Specificity
SD	Standard deviation
SVM	Support Vector Machine
TME	Tumor microenvironment
VEGFA	Vascular endothelial growth factor-A
