# Supplementary material for: Multi-Phasic CECT Peritumoral Radiomics Predict Treatment Response to Bevacizumab-Based Chemotherapy in RAS-Mutated Colorectal Liver Metastases
Source: Bioengineering (Basel). 2026 Jan 24;13(2):137. doi: 10.3390/bioengineering13020137 (PMC12938704; doi:10.3390/bioengineering13020137)
Supplement: Supplementary file 1 [file bioengineering-13-00137-s001.zip › bioengineering-4016011-supplementary.pdf]

Supplementary Material

1 Supplementary Material

Material S1. Acquisition Equipment

All patients underwent enhanced abdominal CT scans. Patients were examined using 256-slice (Brilliance iCT, Philips Healthcare, the Netherlands) multidetector CT scanners. Parameters used were: tube voltage, 120 keV; tube current, 130 – 250 mAs; collimation, 0.625 – 2.5 mm, slice thickness, 1 – 5 mm; reconstruction interval, 0.625 – 1.25 mm.Contrast agents were injected using a high-pressure pump syringe at a speed of 2.5 – 3.5 mL/s. Portal phase CT scans were performed 55-75 s after injecting contrast agents.

Material S2. Detailed Information on Treatment Regimens

All enrolled patients received bevacizumab-based combination chemotherapy, with bevacizumab administered intravenously at a dose of 5 mg/kg every 2 weeks. The specific standard chemotherapy regimens were individually selected based on the patient's performance status, organ function, and clinical guideline recommendations, as detailed below:

FOLFOX regimen: Oxaliplatin 85 mg/m^2 intravenously (Day 1), leucovorin 400 mg/m^2 intravenously (Day 1), and fluorouracil 400 mg/m^2 intravenously as a bolus injection (Day 1), followed by a continuous intravenous infusion of 2400 mg/m^2 for 46 hours.

FOLFIRI regimen: Irinotecan 180 mg/m^2 intravenously (Day 1), leucovorin 400 mg/m^2 intravenously (Day 1), and fluorouracil 400 mg/m^2 intravenously as a bolus injection (Day 1), followed by a continuous intravenous infusion of 2400 mg/m^2 for 46 hours.

CAPEOX regimen: Capecitabine 1000 mg/m^2 orally (twice daily, Days 1-14) and oxaliplatin 130 mg/m^2 intravenously (Day 1).

The interval of chemotherapy was kept consistent with that of bevacizumab treatment.

Dose adjustments or symptomatic supportive treatment were performed according to adverse reactions (such as myelosuppression, gastrointestinal reactions, hand-foot syndrome, etc.) during treatment. All regimens were continued until disease progression, occurrence of intolerable toxic reactions, or when treatment regimen replacement was deemed necessary based on the patient's condition with the patient's informed consent obtained.

2 Supplementary Figures and Tables

2.1 Supplementary Figures

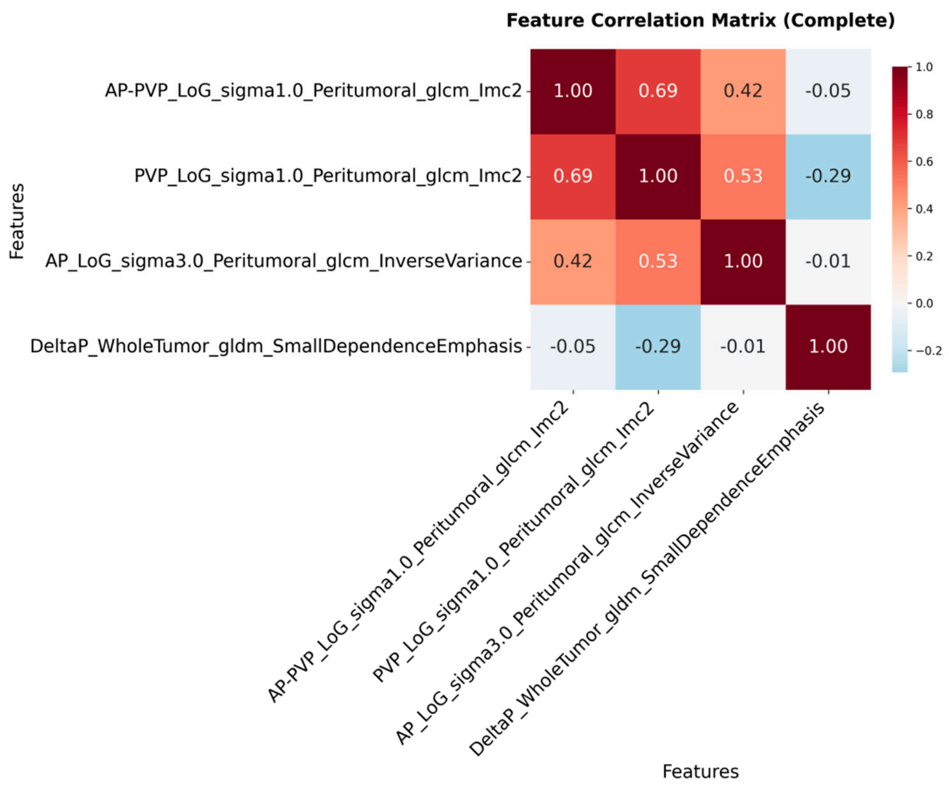

Figure S1. Correlation Matrix of the Final Radiomic Features. Heatmap visualizes the Spearman correlation coefficients of the four radiomic features selected by LASSO regression. The low inter-feature correlations confirm the non-redundancy of the feature set used to build the final Naïve Bayes classifier.

2.2 Supplementary Tables

We evaluated the inter-observer agreement between the two radiologists on a stratified subset of 10 cases (randomly selected to balance responders and non-responders across both training and testing sets).

Table S1. Inter-observer agreement (Dice Similarity Coefficient) of 2D tumor ROI delineation between 2 radiologists in Arterial Phase (AP) and Portal Venous Phase (PVP).

Patient ID	Clinical Status	Dataset	AP DSC	PVP DSC
Patient-06	Responder	Train dataset	0.873	0.926
Patient-09	Responder	Train dataset	0.949	0.949
Patient-13	Responder	Train dataset	0.861	0.894
Patient-17	Non-Responder	Train dataset	0.876	0.895
Patient-20	Non-Responder	Train dataset	0.933	0.934
Patient-21	Non-Responder	Train dataset	0.865	0.885
Patient-04	Responder	Test dataset	0.974	0.961
Patient-09	Responder	Test dataset	0.974	0.939
Patient-16	Non-Responder	Test dataset	0.919	0.930
Patient-18	Non-Responder	Test dataset	0.941	0.942
	**Mean ± SD**		**0.917 ± 0.044**	**0.926 ± 0.026**

Table S2. TRIPOD Checklist for Prediction Model Development and Validation.

Section/Topic	Item	Checklist item	Chapter/Table of the report
**TITLE**	Title	1	Identify the study as developing a prediction model, the target population, and the outcome to be predicted.
**ABSTRACT**	Abstract	2	Provide a summary of objectives, study design, setting, participants, predictors, outcomes, analyses, results, and conclusions.
**INTRODUCTION**	Background	3a-b	Explain the clinical context, rationale, target population, and intended purpose of the model.
	Objectives	4	Specify the study objectives.
**METHODS**	Data	5a	Describe the sources of data (retrospective analysis of prospectively collected data from two centers).
		5b	Specify the dates of participant data collection (1 Jan 2017 to 31 Jun 2025).
	Participants	6a	Specify key elements of the study setting (Nanfang Hospital and Zhujiang Hospital).
		6b	Describe the eligibility criteria for study participants.
	Outcome	8a	Clearly define the outcome being predicted (Bevacizumab resistance, based on RECIST 1.1).
	Predictors	9b	Clearly define all predictors (peri-tumoral radiomic features from multi-phasic CT).
	Sample size	10	Explain how the study size arrived at.
	Missing data	11	Describe how missing data were handled (by exclusion).
	Analytical methods	12a	Describe how the data were used (split into discovery and validation cohorts).

		12b	Describe how predictors were handled (standardization).
		12c	Specify the type of model, all model-building steps, hyperparameter tuning, and internal validation.
		12e	Specify all measures used to evaluate model performance (AUC, sensitivity, specificity, SHAP).
	Model output	15	Specify how classification thresholds were identified (Youden's J statistic).
	Ethical approval	17	Name the institutional review board and describe consent.
**OPEN SCIENCE**	Protocol	18c	State that a protocol was not prepared.
	Registration	18d	State that the study was not registered.
	Data sharing	18e	Provide details of the availability of the study data.
	Code sharing	18f	Provide details of the availability of the analytical code.
**RESULTS**	Participants	20a	Describe the flow of participants through the study.
		20b	Report on the characteristics of study participants.
	Model development	21	Specify the number of participants and outcome events in each analysis.
	Model specification	22	Provide details of the full prediction model to allow predictions in new individuals.
	Model performance	23a	Report model performance estimates with confidence intervals.
**DISCUSSION**	Interpretation	25	Give an overall interpretation of the main results.

	Limitations	26	Discuss any limitations of the study.
	Usability/Implications	27c	Discuss any next steps for future research and generalizability.

We approximate each tumor nodule as an ellipse, where the major axis is denoted as A and the minor axis as B. We then calculate the equivalent diameter R and sum the equivalent diameters of all tumor nodules (SUM) in each patient to obtain the total diameter of the patient's tumor nodules.

Table S3a. Sum of diameter of tumor nodule in Train Cohort.

Train Cohort	A(cm)	B(cm)	R(cm)	SUM (cm)	mean±SD (cm)
Patient-01	1.53	0.89	1.17	2.41	6.46±5.84
	1.48	1.05	1.25		
Patient-02	1.56	1.18	1.36	1.36	
Patient-03	3.32	1.87	2.49	2.49	
Patient-04	4.43	3.63	4.01	7.70	
	4.50	3.03	3.69		
Patient-05	1.70	1.30	1.49	1.49	
Patient-06	3.81	2.38	3.01	4.73	
	2.02	1.45	1.71		
Patient-07	7.31	5.27	6.21	6.21	
Patient-08	7.68	6.86	7.26	7.26	
Patient-9	2.30	2.03	2.16	7.00	
	1.78	1.51	1.64		
	3.54	2.89	3.20		
Patient-10	3.28	2.38	2.80	5.05	
	2.45	2.06	2.25		
Patient-11	1.20	0.71	0.93	4.70	
	2.07	1.63	1.84		
	1.12	1.03	1.07		
	0.88	0.84	0.86		
Patient-12	1.76	1.68	1.72	5.89	
	1.43	1.35	1.39		
	1.71	1.15	1.40		
	1.69	1.12	1.38		
Patient-13	2.21	1.94	2.07	10.39	
	1.44	1.38	1.41		
	2.30	1.58	1.91		
	1.79	1.59	1.69		
	1.94	1.24	1.55		
	1.82	1.71	1.76		
Patient-14	1.64	1.62	1.63	6.35	
	2.44	1.65	2.01		
	2.90	2.55	2.72		

	10.65	3.15	5.80	
	2.77	2.00	2.35	
	3.80	3.11	3.44	
	1.87	1.64	1.75	
Patient-15	1.95	0.73	1.20	26.04
	6.29	5.31	5.78	
	3.34	2.28	2.76	
	3.35	2.64	2.97	
Patient-16	2.70	2.26	2.47	2.47
	2.33	1.65	1.96	
Patient-17	1.43	0.99	1.19	6.87
	1.88	1.37	1.61	
	2.47	1.79	2.10	
Patient-18	1.06	0.72	0.88	5.42
	5.16	4.00	4.54	5.42
Patient-19	5.09	3.81	4.41	6.30
	2.26	1.58	1.89	6.30
Patient-20	3.49	2.24	2.80	2.80
Patient-21	4.23	1.34	2.38	2.38
	6.29	3.35	4.59	
Patient-22	1.07	1.05	1.06	8.04
	1.27	1.08	1.17	
	1.58	0.94	1.22	
Patient-23	3.29	1.65	2.33	2.33
	6.90	5.38	6.09	
Patient-24	2.28	1.95	2.11	12.45
	5.92	3.05	4.25	
Patient-25	1.66	1.32	1.48	2.91
	1.45	1.41	1.43	
	2.40	1.84	2.10	
	3.36	2.65	2.98	
	2.89	1.92	2.36	
	3.71	2.01	2.73	
	2.86	2.28	2.55	
Patient-26	3.96	2.67	3.25	30.09
	2.68	2.05	2.34	
	4.07	3.08	3.54	
	1.75	1.60	1.67	
	3.19	2.78	2.98	
	4.14	3.09	3.57	

Patient-27	1.14	0.96	1.05	3.40
	2.52	2.19	2.35	
	1.73	1.07	1.36	
	2.02	1.67	1.84	
Patient-28	2.00	1.96	1.98	9.19
	3.19	1.74	2.36	
	2.09	1.32	1.66	
	1.71	1.62	1.66	
Patient-29	1.87	1.80	1.83	5.80
	2.57	2.06	2.30	
Patient-30	2.14	1.21	1.61	1.61

Table S3b. Sum of diameter of tumor nodule in Test Cohort.

Test Cohort	A(cm)	B(cm)	R(cm)	SUM (cm)	mean±SD (cm)
Patient-01	1.86	1.75	1.81	1.81	5.98±4.25
	3.77	2.63	3.15		
Patient-02	2.27	1.60	1.91	6.65	
	1.77	1.43	1.59		
Patient-03	7.71	5.50	6.51	6.51	
Patient-04	0.89	0.71	0.79	2.52	
	1.68	1.78	1.73		
	1.76	1.29	1.50		
	1.82	1.02	1.36		
	0.72	0.70	0.71		
	1.20	1.14	1.17		
	1.22	0.99	1.10		
Patient-05	3.04	2.72	2.87	14.15	
	1.81	1.17	1.46		
	1.93	1.39	1.64		
	1.23	0.94	1.08		
	0.49	0.48	0.49		
	0.78	0.77	0.77		
	1.35	1.10	1.22		
Patient-06	0.91	0.80	0.86	3.48	
	1.68	1.18	1.41		
Patient-07	1.52	1.12	1.30	2.85	
	1.69	1.42	1.55		
	1.03	0.80	0.91		
	0.84	0.66	0.74		
Patient-08	0.68	0.52	0.60	6.50	
	1.54	1.20	1.36		
	0.86	1.02	0.94		
	2.52	1.52	1.96		
	0.90	0.89	0.90		
Patient-09	1.47	1.46	1.46	6.59	
	1.48	1.39	1.43		
	1.20	1.18	1.19		

	1.61	1.59	1.60	
	6.11	4.60	5.30	
Patient-10	4.02	3.62	3.81	13.20
	4.79	3.48	4.08	
	0.92	0.68	0.79	
	0.91	0.78	0.84	
Patient-11	0.82	0.49	0.63	3.91
	0.79	0.51	0.64	
	1.05	0.96	1.01	
Patient-12	2.32	1.63	1.94	1.94

This table provides the baseline characteristic information of 42 enrolled patients (Train Cohort: 30 patients, Test Cohort: 12 patients). It lists key details including the patients' gender, type of cancer, bevacizumab resistance status, age at diagnosis, tumor stage, tumor histology type, RAS gene mutation type, number of metastatic lesions excluding liver metastases, nature of metastatic disease, and chemotherapy regimen. After applying the False Discovery Rate (FDR) correction, no significant differences were found between the training and test sets for any variables.

Table S4. Patient Characteristic Information.

Variables	Overall (N=42)	Train cohort (N=30)	Test cohort (N=12)	P-value	FDR Adjusted p-Value
Sex					
Female	17	11	6	0.498	0.908
Male	25	19	6		
Primary tumor					
Rectal cancer	15	9	6	0.292	0.908
Colon cancer	27	21	6		
Resistance to Bevacizumab?					
No resistance	21	16	5	0.495	0.908
Resistant	21	14	7		
Age of Diagnosis (mean ± SD)	55.90±10.88	56.87±10.89	53.50±11.41	0.897	0.997
Clinical T stage of CRLM					
T3	18	12	6	0.732	0.994
T4	24	18	6		
Clinical N stage of CRLM					
N0-1	31	22	9	1.000	1.000
N2	11	8	3		
Histologic types of tumor					
Poorly differentiated adenocarcinoma	0	0	0		
Moderately-poorly differentiated adenocarcinoma	1	1	0		
	37	26	11	0.795	0.994
Moderately differentiated adenocarcinoma	1	1	0		
Moderately-well differentiated adenocarcinoma	1	1	1		
Well differentiated adenocarcinoma	1	1	0		

Mucinous adenocarcinoma					
RAS gene mutation type					
NRAS	6	3	3	0.389	0.908
KRAS	36	27	9		
The character of metastatic disease					
primary metastatic	39	27	12	0.545	0.908
post-surgical relapse	3	3	0		
Chemotherapy Regimen					
CAPEOX	16	14	2		
FOLFIRI	12	10	2	0.015	0.150
FOLFOX	14	6	8		
